# Redetermination of LaZn_5_ based on single crystal X-ray diffraction data

**DOI:** 10.1107/S1600536811050987

**Published:** 2011-12-03

**Authors:** Igor Oshchapovsky, Oksana Zelinska, Beata Rozdzynska-Kielbik, Volodymyr Pavlyuk

**Affiliations:** a Department of Inorganic Chemistry, Ivan Franko National University of Lviv, Kyryla i Mefodia str. 6, 79005 Lviv, Ukraine; b Institute of Chemistry, Environmental Protection and Biotechnology, Jan Dlugosz University, Armii Krajowej Ave. 13/15, 42-200 Czestochowa, Poland

## Abstract

The crystal structure of the already known binary title compound LaZn_5_ (lanthanum penta­zinc) (space group *P*6/*mmm*, Pearson symbol *hP6*, CaCu_5_ structure type) has been redetermined from single-crystal X-ray diffraction data. In contrast to previous determinations based on X-ray powder data [Nowotny (1942). *Z. Metallkd.* **34**, 247–253; de Negri *et al.* (2008). *Inter­metallics*, **16**, 168–178], where unit-cell parameters and assignment of the structure type were reported, the present study reveals anisotropic displacement parameters for all atoms. The crystal structure consists of three crytallographically distinct atoms. The La atom (Wyckoff site 1*a*, site symmetry 6/*mmm*) is surrounded by 18 Zn atoms and two La atoms. The coordination polyhedron around one of the Zn atoms (Wyckoff site 2*c*, site symmetry -6*m*2) is an icosa­hedron made up from three La and nine Zn atoms. The other Zn atom (Wyckoff site 3*g*, site symmetry *mmm*) is surrounded by four La and eight Zn atoms. Bonding between atoms is explored by means of the TB–LMTO–ASA (tight-binding linear muffin-tin orbital atomic spheres approximation) program package. The positive charge density is localized around La atoms, and the negative charge density is around Zn atoms, with weak covalent bonding between the latter.

## Related literature

For previous structural studies of the title compound, see: de Negri *et al.* (2008 ▶); Nowotny (1942 ▶). For general background, see: Andersen *et al.* (1986 ▶); Berche *et al.* (2009 ▶); Oshchapovsky *et al.* (2011**a* ▶,b* ▶); Pavlyuk *et al.* (2009 ▶); Zelinska *et al.* (2004 ▶).

## Experimental

### 

#### Crystal data

LaZn_5_
*M* *_r_* = 465.86
Hexagonal, 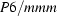

*a* = 5.4654 (17) Å
*c* = 4.2574 (15) Å
*V* = 110.13 (6) Å^3^
*Z* = 1
Mo *K*α radiation
μ = 36.05 mm^−1^
*T* = 296 K
0.04 × 0.02 × 0.02 mm

#### Data collection

Bruker APEXII CCD diffractometer
Absorption correction: multi-scan (*SADABS*; Bruker, 2004 ▶) *T*_min_ = 0.410, *T*_max_ = 0.478
1123 measured reflections
73 independent reflections
62 reflections with *I* > 2σ(*I*)
*R*_int_ = 0.069

#### Refinement

*R*[*F* ^2^ > 2σ(*F* ^2^)] = 0.018
*wR*(*F* ^2^) = 0.037
*S* = 1.17
73 reflections
9 parameters
Δρ_max_ = 1.11 e Å^−3^
Δρ_min_ = −0.71 e Å^−3^

### 

Data collection: *APEX2* (Bruker, 2004 ▶); cell refinement: *SAINT* (Bruker, 2004 ▶); data reduction: *SAINT*; program(s) used to solve structure: *SHELXS97* (Sheldrick, 2008 ▶); program(s) used to refine structure: *SHELXL97* (Sheldrick, 2008 ▶); molecular graphics: *DIAMOND* (Brandenburg, 2006 ▶) and *VESTA* (Momma & Izumi, 2008 ▶); software used to prepare material for publication: *publCIF* (Westrip, 2010 ▶).

## Supplementary Material

Crystal structure: contains datablock(s) I, global. DOI: 10.1107/S1600536811050987/wm2565sup1.cif

Structure factors: contains datablock(s) I. DOI: 10.1107/S1600536811050987/wm2565Isup2.hkl

Additional supplementary materials:  crystallographic information; 3D view; checkCIF report

## Figures and Tables

**Fig. 1.**
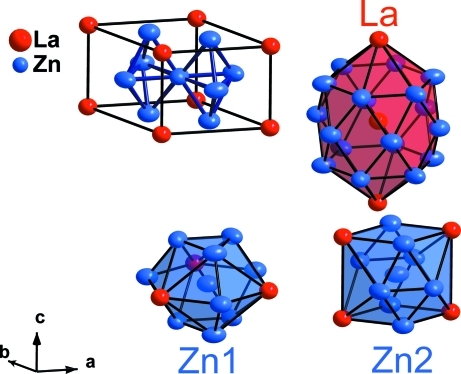
Projection of the unit cell, coordination polyhedra of the atoms and the covalent bonds in the LaZn5 compound. Atoms are given with their anisotropic displacement ellipsoids at the 99.99% probability level.

**Fig. 2.**
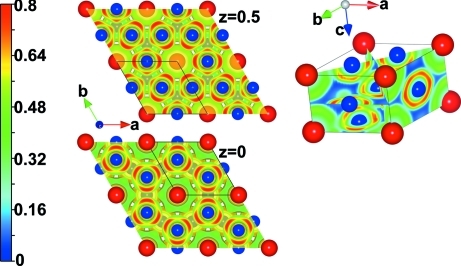
Isosurfaces of ELF drawn at the level 0.3 at z=0 and z=0.5 and sections for the LaZn5 compound.

**Fig. 3.**
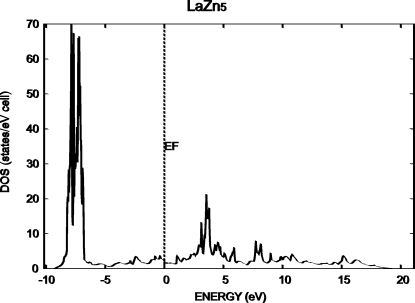
Density of states plot for the LaZn5 compound.

## supplementary crystallographic information

### Comment

This paper is part of a systematic investigation of binary *RE*—Zn (Oshchapovsky *et al.*, 2011*a*; Zelinska *et al.*, 2004) and ternary *RE*—Zn—*M* systems (where *RE* is a rare earth metal and *M* is a *p*-element of group IV) (Oshchapovsky *et al.*, 2011*b*; Pavlyuk *et al.*, 2009). The binary La—Zn system is not completely investigated yet (Berche *et al.*, 2009), especially with respect to the structure of the compound LaZn_4_. In order to determine its crystal structure a sample with the same composition was synthesized. However, this sample was prepared under non-equilibrium conditions and phase analysis from X-ray powder data revealed the presence of LaZn_5_, LaZn_2_, trace amounts of LaZn and strong reflections of unknown phase(s). The lattice parameters of the title LaZn_5_ phase were determined for the first time based on X-ray powder diffraction data (Nowotny, 1942). Previous authors (Nowotny, 1942; de Negri *et al.*, 2008) also assigned the structure type. However, a complete crystal structure determination including anisotropic displacement parameters was not carried out before. Therefore a high-quality single-crystal of LaZn_5_ was selected and the results of the full structure determination are presented in this paper.

The crystal structure consists of three crytallographically distinct atoms. La1 (Wyckoff site 1*a*, site symmetry 6/*mmm*) is surrounded by 18 Zn atoms and 2 La atoms. The coordination polyhedron around Zn1 atom (Wyckoff site 2*c*, site symmetry -6*m*2) is an icosahedron formed by 3 La and 9 Zn atoms. Zn2 (Wyckoff site 3*g*, site symmetry *mmm*) is surrounded by 4 La and 8 Zn atoms. The projection of the LaZn_5_ unit cell is given in Fig. 1. The thermal displacement of the lanthanum atom is almost isotropic. The thermal ellipsoids of the Zn1 atoms are oblate along the c axis. The thermal ellipsoids of the Zn2 atoms are extended along the a and b axes due to the largest space for displacement in this direction (the distances of corresponding atoms to each other and to La atoms are larger than for Zn1 atoms).

The electronic structure of LaZn_5_ was calculated using the TB-LMTO-ASA package (Andersen *et al.*, 1986). The dominant type of bonding in this compound is metallic. The La atoms donate their electrons to the Zn atoms. Therefore positive charge density can be observed around the rare earth atom and negative charge density is around the transition metal atoms. This fact, together with significant electron density (~0.4 e/Å^3^) and significant ELF density (~0.4) between Zn atoms, confirms the weak covalent bonding between them. In other words, an ion–metallic bonding between La and Zn atoms and a covalent–metallic bonding between Zn atoms is evident (Figure 2). A similar way of bond formation is also observed for LaZn_12.37_ (Oshchapovsky *et al.*, 2011*a*) and La_5_Zn_2_Sn (Oshchapovsky *et al.*, 2011*b*) which were investigated previously. The density of states (DOS) plot confirms a metallic-type of conductivity of the title compound (Figure 3), and it is rather similar to the DOS plot for the LaZn_12.37_ compound.

### Experimental

A small irregularly shaped single crystal of LaZn_5_ was selected by mechanical fragmentation of a sample with nominal composition LaZn_4_. The sample was prepared by mixing stoichiometric amounts of Zn and LaZn powders with subsequent pressing into a pellet. This pellet was sealed in an evacuated silica ampoule and annealed in a resistance furnace at 873 K for 30 days and subsequently quenched in cold water. No reaction between the alloy and the silica container was observed.

### Refinement

The highest peak of 1.11 e/Å^3^ is at (0; 0; 0.1905) and 0.81 Å away from the La1 atom. The deepest hole -0.71 e/Å^3^ is at (0; 0; 1/2) and 2.13 Å away from the same atom.

### Crystal data

**Table:**

LaZn_5_	*D*_x_ = 7.024 Mg m^−^^3^
*M**_r_* = 465.86	Mo *K*α radiation, λ = 0.71073 Å
Hexagonal, *P*6/*m**m**m*	Cell parameters from 1123 reflections
Hall symbol: -P 6 2	θ = 4.3–27.5°
*a* = 5.4654 (17) Å	µ = 36.05 mm^−^^1^
*c* = 4.2574 (15) Å	*T* = 296 K
*V* = 110.13 (6) Å^3^	Irregular shape, metallic grey
*Z* = 1	0.04 × 0.02 × 0.02 mm
*F*(000) = 207	

### Data collection

**Table:**

Bruker APEXII CCD diffractometer	73 independent reflections
Radiation source: fine-focus sealed tube	62 reflections with *I* > 2σ(*I*)
graphite	*R*_int_ = 0.069
φ and ω scans	θ_max_ = 27.5°, θ_min_ = 4.3°
Absorption correction: multi-scan (*SADABS*; Bruker, 2004)	*h* = −6→7
*T*_min_ = 0.410, *T*_max_ = 0.478	*k* = −6→7
1123 measured reflections	*l* = −5→5

### Refinement

**Table:**

Refinement on *F*^2^	Primary atom site location: structure-invariant direct methods
Least-squares matrix: full	Secondary atom site location: difference Fourier map
*R*[*F*^2^ > 2σ(*F*^2^)] = 0.018	*w* = 1/[σ^2^(*F*_o_^2^) + (0.*P*)^2^ + 0.1413*P*] where *P* = (*F*_o_^2^ + 2*F*_c_^2^)/3
*wR*(*F*^2^) = 0.037	(Δ/σ)_max_ < 0.001
*S* = 1.17	Δρ_max_ = 1.11 e Å^−^^3^
73 reflections	Δρ_min_ = −0.71 e Å^−^^3^
9 parameters	Extinction correction: *SHELXL97* (Sheldrick, 2008), Fc^*^=kFc[1+0.001xFc^2^λ^3^/sin(2θ)]^-1/4^
0 restraints	Extinction coefficient: 0.022 (4)
0 constraints	

### Special details

**Table:**

Geometry. All e.s.d.'s (except the e.s.d. in the dihedral angle between two l.s. planes) are estimated using the full covariance matrix. The cell e.s.d.'s are taken into account individually in the estimation of e.s.d.'s in distances, angles and torsion angles; correlations between e.s.d.'s in cell parameters are only used when they are defined by crystal symmetry. An approximate (isotropic) treatment of cell e.s.d.'s is used for estimating e.s.d.'s involving l.s. planes.
Refinement. Refinement of *F*^2^ against ALL reflections. The weighted *R*-factor *wR* and goodness of fit *S* are based on *F*^2^, conventional *R*-factors *R* are based on *F*, with *F* set to zero for negative *F*^2^. The threshold expression of *F*^2^ > σ(*F*^2^) is used only for calculating *R*-factors(gt) *etc*. and is not relevant to the choice of reflections for refinement. *R*-factors based on *F*^2^ are statistically about twice as large as those based on *F*, and *R*- factors based on ALL data will be even larger.

### Fractional atomic coordinates and isotropic or equivalent isotropic displacement parameters (Å^2^)

**Table:**

	*x*	*y*	*z*	*U*_iso_*/*U*_eq_	
La1	0.0000	0.0000	0.0000	0.0099 (4)	
Zn1	0.3333	0.6667	0.0000	0.0124 (4)	
Zn2	0.5000	1.0000	0.5000	0.0121 (4)	

### Atomic displacement parameters (Å^2^)

**Table:**

	*U*^11^	*U*^22^	*U*^33^	*U*^12^	*U*^13^	*U*^23^
La1	0.0106 (4)	0.0106 (4)	0.0085 (6)	0.0053 (2)	0.000	0.000
Zn1	0.0146 (5)	0.0146 (5)	0.0081 (8)	0.0073 (3)	0.000	0.000
Zn2	0.0162 (5)	0.0104 (6)	0.0079 (6)	0.0052 (3)	0.000	0.000

### Geometric parameters (Å, °)

**Table:**

La1—Zn1^i^	3.1554 (10)	Zn1—Zn2^xiii^	2.6496 (7)
La1—Zn1^ii^	3.1554 (10)	Zn1—Zn1^i^	3.1554 (10)
La1—Zn1^iii^	3.1554 (10)	Zn1—La1^xiv^	3.1555 (10)
La1—Zn1	3.1555 (10)	Zn1—La1^xv^	3.1555 (10)
La1—Zn1^iv^	3.1555 (10)	Zn1—Zn1^xvi^	3.1555 (10)
La1—Zn1^v^	3.1555 (10)	Zn1—Zn1^v^	3.1555 (10)
La1—Zn2^vi^	3.4640 (8)	Zn2—Zn1^xvii^	2.6496 (7)
La1—Zn2^vii^	3.4640 (8)	Zn2—Zn1^xvi^	2.6496 (7)
La1—Zn2^viii^	3.4640 (8)	Zn2—Zn1^xviii^	2.6496 (7)
La1—Zn2^ix^	3.4640 (8)	Zn2—Zn2^xix^	2.7327 (8)
La1—Zn2^iv^	3.4640 (8)	Zn2—Zn2^viii^	2.7327 (8)
La1—Zn2^x^	3.4640 (8)	Zn2—Zn2^xx^	2.7327 (8)
Zn1—Zn2^xi^	2.6496 (7)	Zn2—Zn2^vi^	2.7327 (8)
Zn1—Zn2	2.6496 (7)	Zn2—La1^xxi^	3.4640 (8)
Zn1—Zn2^viii^	2.6496 (7)	Zn2—La1^xiv^	3.4640 (8)
Zn1—Zn2^xii^	2.6496 (7)	Zn2—La1^xv^	3.4640 (8)
Zn1—Zn2^vi^	2.6496 (7)	Zn2—La1^xxii^	3.4640 (8)
			
Zn1^i^—La1—Zn1^ii^	120.0	Zn2^xiii^—Zn1—La1^xv^	72.679 (6)
Zn1^i^—La1—Zn1^iii^	180.0	Zn1^i^—Zn1—La1^xv^	60.0
Zn1^ii^—La1—Zn1^iii^	60.0	La1^xiv^—Zn1—La1^xv^	120.0
Zn1^i^—La1—Zn1	60.0	Zn2^xi^—Zn1—La1	72.679 (6)
Zn1^ii^—La1—Zn1	180.0	Zn2—Zn1—La1	126.545 (13)
Zn1^iii^—La1—Zn1	120.0	Zn2^viii^—Zn1—La1	72.679 (6)
Zn1^i^—La1—Zn1^iv^	60.0	Zn2^xii^—Zn1—La1	126.545 (13)
Zn1^ii^—La1—Zn1^iv^	60.0	Zn2^vi^—Zn1—La1	72.679 (5)
Zn1^iii^—La1—Zn1^iv^	120.0	Zn2^xiii^—Zn1—La1	72.679 (6)
Zn1—La1—Zn1^iv^	120.0	Zn1^i^—Zn1—La1	60.0
Zn1^i^—La1—Zn1^v^	120.0	La1^xiv^—Zn1—La1	120.0
Zn1^ii^—La1—Zn1^v^	120.0	La1^xv^—Zn1—La1	120.0
Zn1^iii^—La1—Zn1^v^	60.0	Zn2^xi^—Zn1—Zn1^xvi^	107.321 (5)
Zn1—La1—Zn1^v^	60.0	Zn2—Zn1—Zn1^xvi^	53.455 (13)
Zn1^iv^—La1—Zn1^v^	180.0	Zn2^viii^—Zn1—Zn1^xvi^	107.321 (6)
Zn1^i^—La1—Zn2^vi^	46.905 (10)	Zn2^xii^—Zn1—Zn1^xvi^	53.455 (13)
Zn1^ii^—La1—Zn2^vi^	133.095 (10)	Zn2^vi^—Zn1—Zn1^xvi^	107.321 (6)
Zn1^iii^—La1—Zn2^vi^	133.095 (10)	Zn2^xiii^—Zn1—Zn1^xvi^	107.321 (6)
Zn1—La1—Zn2^vi^	46.906 (10)	Zn1^i^—Zn1—Zn1^xvi^	120.0
Zn1^iv^—La1—Zn2^vi^	90.0	La1^xiv^—Zn1—Zn1^xvi^	60.0
Zn1^v^—La1—Zn2^vi^	90.0	La1^xv^—Zn1—Zn1^xvi^	60.0
Zn1^i^—La1—Zn2^vii^	133.095 (10)	La1—Zn1—Zn1^xvi^	180.0
Zn1^ii^—La1—Zn2^vii^	46.905 (10)	Zn2^xi^—Zn1—Zn1^v^	53.455 (13)
Zn1^iii^—La1—Zn2^vii^	46.905 (10)	Zn2—Zn1—Zn1^v^	107.321 (5)
Zn1—La1—Zn2^vii^	133.094 (10)	Zn2^viii^—Zn1—Zn1^v^	53.455 (13)
Zn1^iv^—La1—Zn2^vii^	90.0	Zn2^xii^—Zn1—Zn1^v^	107.321 (6)
Zn1^v^—La1—Zn2^vii^	90.0	Zn2^vi^—Zn1—Zn1^v^	107.321 (6)
Zn2^vi^—La1—Zn2^vii^	180.0	Zn2^xiii^—Zn1—Zn1^v^	107.321 (6)
Zn1^i^—La1—Zn2^viii^	90.0	Zn1^i^—Zn1—Zn1^v^	120.0
Zn1^ii^—La1—Zn2^viii^	133.094 (9)	La1^xiv^—Zn1—Zn1^v^	60.0
Zn1^iii^—La1—Zn2^viii^	90.0	La1^xv^—Zn1—Zn1^v^	180.0
Zn1—La1—Zn2^viii^	46.906 (10)	La1—Zn1—Zn1^v^	60.0
Zn1^iv^—La1—Zn2^viii^	133.094 (10)	Zn1^xvi^—Zn1—Zn1^v^	120.0
Zn1^v^—La1—Zn2^viii^	46.906 (10)	Zn1—Zn2—Zn1^xvii^	180.0
Zn2^vi^—La1—Zn2^viii^	46.463 (8)	Zn1—Zn2—Zn1^xvi^	73.09 (3)
Zn2^vii^—La1—Zn2^viii^	133.537 (8)	Zn1^xvii^—Zn2—Zn1^xvi^	106.91 (3)
Zn1^i^—La1—Zn2^ix^	90.0	Zn1—Zn2—Zn1^xviii^	106.91 (3)
Zn1^ii^—La1—Zn2^ix^	46.906 (9)	Zn1^xvii^—Zn2—Zn1^xviii^	73.09 (3)
Zn1^iii^—La1—Zn2^ix^	90.0	Zn1^xvi^—Zn2—Zn1^xviii^	180.0
Zn1—La1—Zn2^ix^	133.094 (10)	Zn1—Zn2—Zn2^xix^	121.043 (10)
Zn1^iv^—La1—Zn2^ix^	46.906 (10)	Zn1^xvii^—Zn2—Zn2^xix^	58.958 (10)
Zn1^v^—La1—Zn2^ix^	133.094 (10)	Zn1^xvi^—Zn2—Zn2^xix^	58.958 (10)
Zn2^vi^—La1—Zn2^ix^	133.537 (8)	Zn1^xviii^—Zn2—Zn2^xix^	121.042 (10)
Zn2^vii^—La1—Zn2^ix^	46.463 (8)	Zn1—Zn2—Zn2^viii^	58.957 (10)
Zn2^viii^—La1—Zn2^ix^	180.0	Zn1^xvii^—Zn2—Zn2^viii^	121.042 (11)
Zn1^i^—La1—Zn2^iv^	46.906 (9)	Zn1^xvi^—Zn2—Zn2^viii^	121.042 (10)
Zn1^ii^—La1—Zn2^iv^	90.0	Zn1^xviii^—Zn2—Zn2^viii^	58.958 (10)
Zn1^iii^—La1—Zn2^iv^	133.094 (9)	Zn2^xix^—Zn2—Zn2^viii^	180.0
Zn1—La1—Zn2^iv^	90.0	Zn1—Zn2—Zn2^xx^	121.043 (10)
Zn1^iv^—La1—Zn2^iv^	46.906 (10)	Zn1^xvii^—Zn2—Zn2^xx^	58.957 (10)
Zn1^v^—La1—Zn2^iv^	133.094 (10)	Zn1^xvi^—Zn2—Zn2^xx^	58.957 (10)
Zn2^vi^—La1—Zn2^iv^	46.463 (8)	Zn1^xviii^—Zn2—Zn2^xx^	121.043 (10)
Zn2^vii^—La1—Zn2^iv^	133.537 (8)	Zn2^xix^—Zn2—Zn2^xx^	60.0
Zn2^viii^—La1—Zn2^iv^	86.189 (19)	Zn2^viii^—Zn2—Zn2^xx^	120.0
Zn2^ix^—La1—Zn2^iv^	93.811 (19)	Zn1—Zn2—Zn2^vi^	58.957 (10)
Zn1^i^—La1—Zn2^x^	133.094 (9)	Zn1^xvii^—Zn2—Zn2^vi^	121.043 (10)
Zn1^ii^—La1—Zn2^x^	90.0	Zn1^xvi^—Zn2—Zn2^vi^	121.043 (10)
Zn1^iii^—La1—Zn2^x^	46.906 (9)	Zn1^xviii^—Zn2—Zn2^vi^	58.957 (10)
Zn1—La1—Zn2^x^	90.0	Zn2^xix^—Zn2—Zn2^vi^	120.0
Zn1^iv^—La1—Zn2^x^	133.094 (10)	Zn2^viii^—Zn2—Zn2^vi^	60.0
Zn1^v^—La1—Zn2^x^	46.906 (10)	Zn2^xx^—Zn2—Zn2^vi^	180.0
Zn2^vi^—La1—Zn2^x^	133.537 (8)	Zn1—Zn2—La1^xxi^	119.585 (15)
Zn2^vii^—La1—Zn2^x^	46.463 (8)	Zn1^xvii^—Zn2—La1^xxi^	60.416 (15)
Zn2^viii^—La1—Zn2^x^	93.811 (19)	Zn1^xvi^—Zn2—La1^xxi^	119.585 (15)
Zn2^ix^—La1—Zn2^x^	86.189 (19)	Zn1^xviii^—Zn2—La1^xxi^	60.415 (15)
Zn2^iv^—La1—Zn2^x^	180.0	Zn2^xix^—Zn2—La1^xxi^	66.768 (4)
Zn2^xi^—Zn1—Zn2	145.358 (11)	Zn2^viii^—Zn2—La1^xxi^	113.232 (4)
Zn2^xi^—Zn1—Zn2^viii^	106.91 (3)	Zn2^xx^—Zn2—La1^xxi^	113.232 (4)
Zn2—Zn1—Zn2^viii^	62.09 (2)	Zn2^vi^—Zn2—La1^xxi^	66.768 (4)
Zn2^xi^—Zn1—Zn2^xii^	62.09 (2)	Zn1—Zn2—La1^xiv^	60.415 (15)
Zn2—Zn1—Zn2^xii^	106.91 (3)	Zn1^xvii^—Zn2—La1^xiv^	119.584 (15)
Zn2^viii^—Zn1—Zn2^xii^	145.358 (11)	Zn1^xvi^—Zn2—La1^xiv^	60.415 (15)
Zn2^xi^—Zn1—Zn2^vi^	145.358 (11)	Zn1^xviii^—Zn2—La1^xiv^	119.585 (15)
Zn2—Zn1—Zn2^vi^	62.09 (2)	Zn2^xix^—Zn2—La1^xiv^	113.232 (5)
Zn2^viii^—Zn1—Zn2^vi^	62.09 (2)	Zn2^viii^—Zn2—La1^xiv^	66.768 (4)
Zn2^xii^—Zn1—Zn2^vi^	145.358 (11)	Zn2^xx^—Zn2—La1^xiv^	66.768 (5)
Zn2^xi^—Zn1—Zn2^xiii^	62.09 (2)	Zn2^vi^—Zn2—La1^xiv^	113.232 (4)
Zn2—Zn1—Zn2^xiii^	145.358 (11)	La1^xxi^—Zn2—La1^xiv^	180.0
Zn2^viii^—Zn1—Zn2^xiii^	145.358 (11)	Zn1—Zn2—La1^xv^	60.415 (15)
Zn2^xii^—Zn1—Zn2^xiii^	62.09 (2)	Zn1^xvii^—Zn2—La1^xv^	119.585 (15)
Zn2^vi^—Zn1—Zn2^xiii^	106.91 (3)	Zn1^xvi^—Zn2—La1^xv^	60.416 (15)
Zn2^xi^—Zn1—Zn1^i^	107.321 (6)	Zn1^xviii^—Zn2—La1^xv^	119.584 (15)
Zn2—Zn1—Zn1^i^	107.321 (6)	Zn2^xix^—Zn2—La1^xv^	66.768 (4)
Zn2^viii^—Zn1—Zn1^i^	107.321 (6)	Zn2^viii^—Zn2—La1^xv^	113.232 (4)
Zn2^xii^—Zn1—Zn1^i^	107.321 (6)	Zn2^xx^—Zn2—La1^xv^	113.232 (4)
Zn2^vi^—Zn1—Zn1^i^	53.455 (13)	Zn2^vi^—Zn2—La1^xv^	66.768 (4)
Zn2^xiii^—Zn1—Zn1^i^	53.455 (13)	La1^xxi^—Zn2—La1^xv^	75.84 (3)
Zn2^xi^—Zn1—La1^xiv^	72.679 (6)	La1^xiv^—Zn2—La1^xv^	104.16 (3)
Zn2—Zn1—La1^xiv^	72.679 (6)	Zn1—Zn2—La1^xxii^	119.585 (15)
Zn2^viii^—Zn1—La1^xiv^	72.679 (6)	Zn1^xvii^—Zn2—La1^xxii^	60.415 (15)
Zn2^xii^—Zn1—La1^xiv^	72.679 (6)	Zn1^xvi^—Zn2—La1^xxii^	119.584 (15)
Zn2^vi^—Zn1—La1^xiv^	126.545 (13)	Zn1^xviii^—Zn2—La1^xxii^	60.416 (15)
Zn2^xiii^—Zn1—La1^xiv^	126.545 (13)	Zn2^xix^—Zn2—La1^xxii^	113.232 (4)
Zn1^i^—Zn1—La1^xiv^	180.0	Zn2^viii^—Zn2—La1^xxii^	66.768 (4)
Zn2^xi^—Zn1—La1^xv^	126.545 (13)	Zn2^xx^—Zn2—La1^xxii^	66.768 (4)
Zn2—Zn1—La1^xv^	72.679 (6)	Zn2^vi^—Zn2—La1^xxii^	113.232 (4)
Zn2^viii^—Zn1—La1^xv^	126.545 (13)	La1^xxi^—Zn2—La1^xxii^	104.16 (3)
Zn2^xii^—Zn1—La1^xv^	72.679 (6)	La1^xiv^—Zn2—La1^xxii^	75.84 (3)
Zn2^vi^—Zn1—La1^xv^	72.679 (6)	La1^xv^—Zn2—La1^xxii^	180.0

Symmetry codes: (i) −*x*+1, −*y*+1, −*z*; (ii) −*x*, −*y*, −*z*; (iii) *x*−1, *y*−1, *z*; (iv) *x*, *y*−1, *z*; (v) −*x*, −*y*+1, −*z*; (vi) *x*−*y*+1, *x*, *z*; (vii) *x*−*y*, *x*−1, *z*−1; (viii) −*y*+1, *x*−*y*+1, *z*; (ix) −*y*+1, *x*−*y*, *z*−1; (x) *x*−1, *y*−1, *z*−1; (xi) −*y*+1, *x*−*y*+1, *z*−1; (xii) *x*, *y*, *z*−1; (xiii) *x*−*y*+1, *x*, *z*−1; (xiv) *x*, *y*+1, *z*; (xv) *x*+1, *y*+1, *z*; (xvi) −*x*+1, −*y*+2, −*z*; (xvii) −*x*+1, −*y*+2, −*z*+1; (xviii) *x*, *y*, *z*+1; (xix) −*y*+2, *x*−*y*+2, *z*; (xx) *x*−*y*+1, *x*+1, *z*; (xxi) *x*+1, *y*+1, *z*+1; (xxii) *x*, *y*+1, *z*+1.
